# Development of Embryonic Market Squid, *Doryteuthis opalescens*, under Chronic Exposure to Low Environmental pH and [O_2_]

**DOI:** 10.1371/journal.pone.0167461

**Published:** 2016-12-09

**Authors:** Michael O. Navarro, Garfield T. Kwan, Olga Batalov, Chelsea Y. Choi, N. Tessa Pierce, Lisa A. Levin

**Affiliations:** 1 Integrative Oceanography Division, Scripps Institution of Oceanography, UCSD, La Jolla, California, United States of America; 2 Center for Marine Biodiversity and Conservation, Scripps Institution of Oceanography, UCSD, La Jolla, California, United States of America; 3 Marine Biology Research Division, Scripps Institution of Oceanography, UCSD, La Jolla, California, United States of America; 4 Division of Biological Science, UCSD, La Jolla, California, United States of America; 5 Biology Department, University of Rochester, Rochester, New York, United States of America; Helmholtz-Zentrum fur Ozeanforschung Kiel, GERMANY

## Abstract

The market squid, *Doryteuthis opalescens*, is an important forage species for the inshore ecosystems of the California Current System. Due to increased upwelling and expansion of the oxygen minimum zone in the California Current Ecosystem, the inshore environment is expected to experience lower pH and [O_2_] conditions in the future, potentially impacting the development of seafloor-attached encapsulated embryos. To understand the consequences of this co-occurring environmental pH and [O_2_] stress for *D*. *opalescens* encapsulated embryos, we performed two laboratory experiments. In Experiment 1, embryo capsules were chronically exposed to a treatment of higher (normal) pH (7.93) and [O_2_] (242 μM) or a treatment of low pH (7.57) and [O_2_] (80 μM), characteristic of upwelling events and/or La Niña conditions. The low pH and low [O_2_] treatment extended embryo development duration by 5–7 days; embryos remained at less developed stages more often and had 54.7% smaller statolith area at a given embryo size. Importantly, the embryos that did develop to mature embryonic stages grew to sizes that were similar (non-distinct) to those exposed to the high pH and high [O_2_] treatment. In Experiment 2, we exposed encapsulated embryos to a single stressor, low pH (7.56) or low [O_2_] (85 μM), to understand the importance of environmental pH and [O_2_] rising and falling together for squid embryogenesis. Embryos in the low pH only treatment had smaller yolk reserves and bigger statoliths compared to those in low [O_2_] only treatment. These results suggest that *D*. *opalescens* developmental duration and statolith size are impacted by exposure to environmental [O_2_] and pH (*p*CO_2_) and provide insight into embryo resilience to these effects.

## Introduction

Invertebrate embryos have several mechanisms to maintain stable development in response to natural environmental variability, including robust developmental programs and alternative developmental pathways [1]. However, anthropogenic climate change may expose organisms to more extreme environmental conditions during embryogenesis, pushing them beyond their physiological limits [1]. The surface water [O_2_] of the ocean is expected to decline by 1% to 7% globally in over the next century [2] and hypoxic oxygen isopleths have already shoaled by as much as 90 m over the last 25 years in the Southern California Bight with oxygen declines accelerated at inshore regions [3–6]. In addition, the surface water pH of the California Current System has declined by 0.1 units since the start of the industrial revolution [7, 8]. These conditions are of particular concern for commercially valuable species that attach eggs to the sea floor, such as the market squid *Doryteuthis opalescens*. Currently, the sandy La Jolla, CA shelf where *D*. *opalescens* embryos develop is periodically exposed to lower, presumably more stressful, environmental pH and [O_2_] during upwelling and La Niña events, resulting in habitat pH that ranges from 7.65 to 8.10 and [O_2_] that ranges from 70–240 μM [9]. By 2060, pH is expected to decrease by 0.11–0.13 units at the 30-m embryo habitat of *D*. *opalescens* within the Southern California Bight [8]. As *D*. *opalescens* embryos are attached to the seafloor and cannot avoid exposure to intruding low oxygen and low pH waters, they may be vulnerable to secular, climate-driven changes in pH and [O_2_].

Loliginid embryos are dependent on aerobic metabolism and can be negatively affected by low [O_2_] [10–12]. Developing *D*. *opalescens* embryos have a finite energy reserve (*i*.*e*. yolk) for embryogenesis, regulation of internal pH, and the energetically costly excretion of metabolic wastes [13–15]. We define embryogenesis as the time from the laying of egg capsules to initiation of hatching [16]. Under exposure to low pH, cephalopods have been shown to increase excretion of protons and proton equivalents (e.g. NH_4_^+^) via energy-dependent transporters in order to stabilize extracellular pH [17, 18]. *D*. *opalescens* embryos can be exposed to low [O_2_] and low pH waters for the entire embryogenesis process (*i*.*e*. 3–5 weeks) potentially causing additional energy costs and reducing the scope for aerobic performance [19]. In contrast to the embryo stage, *D*. *opalescens* juvenile and adult life stages are mobile, are not restricted to a fixed energy source, and can increase their available energy by consuming more food (up to 35–80% of their body weight each day) to meet metabolic demands [20].

*D*. *opalescens* statoliths are aragonite structures [21] vital for sensing gravity, balance, movement [22, 23] and sound [24]. Statocyst, embryo, chorion and capsule membranes separate statoliths from the environment; these membranes can decouple statoliths from environmental conditions [25]. Low environmental pH/high *p*CO_2_ is known to affect statolith composition [25, 26] and size for loliginids [27]. In a sister species from the Atlantic Ocean, *Doryteuthis pealeii*, statoliths were found to be slightly smaller with exposure to an environmental pH level of ~7.3 [27].

To assess the effects of chronic exposure to realistic low environmental pH and [O_2_] conditions on *D*. *opalescens* embryos, we analyzed the same laboratory experiments and embryo capsules as described in Navarro et al. 2014. Briefly, the first experiment assessed exposure to combined low [O_2_] and low pH, while the second experiment tested the effects of low [O_2_] as compared with low pH [25]. For this study, we focused on assessing the effects of these environmental conditions on embryo developmental duration, yolk size, and statolith size. For the first experiment we hypothesized *a priori* that embryos exposed to low [O_2_] and low pH would have longer developmental duration and be smaller in size (with larger external yolks) at the same embryonic stage (e.g. dorsal mantle length). For the second experiment, we hypothesized that *D*. *opalescens* would have a morphological response to decoupled pH and [O_2_] treatments. Embryos exposed to the low [O_2_] treatment were expected to have a longer developmental duration, while embryos exposed to the low pH treatment were expected to be smaller in size [27–28]. For both experiments, we hypothesized that statolith development in *D*. *opalescens* is coupled to and can be affected by environmental pH and [O_2_].

## Materials and Methods

Newly collected *D*. *opalescens* embryo capsules (California Department of Fish and Wildlife permit #7230) were reared in two separate laboratory experiments using the Multiple Stressor Experimental Aquarium at Scripps (MSEAS) [29] as described in Navarro et al. 2014 [25]. MSEAS used an open-water system, manipulating seawater treatments in each tank independently. For each experiment, treatment designation per tank was randomly assigned. *A priori*, each capsule was randomly assigned a tank and a unique position within the tank that maximized its separation from other capsules. Temperature, [O_2_], pH, and alkalinity were measured discretely (S1 Fig) via daily water samples and continuously measured by tidbit temperature loggers and by rotating Aanderaa [O_2_] and Durafet (pH) sensors among tanks. Samples of embryos from this study were taken from unique aliquots from the same capsules described in Navarro et al. 2014 [25].

Here, we define cohorts as squid collected in the same place at the same time. Experiment 1 used two cohorts, specifically squid capsules collected from the field at La Jolla, USA (cohort 1; n = 24; 32.86°N, 117.27°W, 30 m depth) and capsules laid in captivity at Scripps (cohort 2; n = 16) by squid captured off Del Mar, USA (32.96°N, 117.28°W). Experiment 2 used squid capsules (n = 80) collected from La Jolla, USA (32.87°N, 117.25°W). The number of embryos per capsule in Experiment 1 did not differ between field and laboratory-laid cohorts. In contrast, the squid embryos were different between Experiment 1 and 2 (collected two months apart). The number of embryos per capsule of the pooled cohorts from Experiment 1 (165 ± 7.3 SD) was significantly lower by 15% compared to those from Experiment 2 (190 ± 4.9 SD; *F*_1,112_ = 8.585, ***p = 0*.*004***). Before exposure to treatments, embryos within capsules were visually verified to be at pre-gastrulation stages (Stages 1–9 [30]). The Institutional Animal Care and Use Committee did not regulate cephalopod embryos at the time the laboratory experiments were performed.

As described in Navarro et al. 2014 [25], each experiment contained two treatments with two replicates per treatment. Fixed conditions across all experiments were: temperature = 11.3°C, light = 3000 lux (12:12 hour light: dark daily cycle), salinity = 33.4 PSU. Experiment 1 compared high and low [O_2_] and pH levels, as they vary together with a positive correlation in the field [31–33]. The first treatment, termed “high pHOx,” had [O_2_] of 242.0 μM (± 12.7), a pH of 7.93 (± 0.058) and an alkalinity of 2,214.8 μM (± 5.7). The second treatment, termed “low pHOx,” had [O_2_] of 80.4 μM (± 18.7), a pH level of 7.57 (± 0.066) and an alkalinity of 2,215.1 μM (± 6.0). Treatment values in Experiment 1 fell within the range characteristic of upwelling (low pHOx) and relaxation (high pHOx) events in the region at ~30 m depth [32] where squid embryos commonly are observed [9, 12]. In nature, *D*. *opalescens* are exposed to a range of [O_2_] and pH conditions because they can spawn at all times of the year in the Southern California Bight [9]. The low pHOx conditions simulate a present-day, worst-case scenario of [O_2_] and pH exposure. This type of exposure could reasonably occur during conditions with a persistent isopycnal uplift, such as occurring during a La Niña and/or a strong upwelling season [9, 32–33]. Experiment 2 tested [O_2_] and pH each as a single stressor (*i*.*e*. low [O_2_] only or low pH only). This experiment was designed to identify if squid develop differently when [O_2_] and pH levels are de-coupled from one another, to assess whether or not [O_2_] and pH coupling impacts development. The “low pH” only treatment pH level was 7.56 (± 0.028) with alkalinity of 2,242.7 μM (± 6.5) and [O_2_] at 241.4 μM (± 8.4). Whereas the “low [O_2_]” only treatment had an [O_2_] of 84.7 μM (± 10.6), with a pH level of 7.92 (± 0.054) and alkalinity of 2,240.5 μM (± 5.0). In both experiments, pH was controlled by manipulating *p*CO_2_, and alkalinity remained relatively constant (total alkalinity range = 2214–2244 μM) [25, 29]. Treatment conditions (O_2_ and pH) were distinct in each experiment, however, seawater temperature, and alkalinity did not differ between treatments [25]. A Kruskal-Wallis test revealed no tank effects in Experiment 1 other than those associated with treatments (i.e. pH and [O_2_] were different between low pHOx and high pHOx tanks; S1 Fig). However, statistically significant differences in non-treatment factors (TA and temperature) were found among tanks in Experiment 2 using Kruskal-Wallis tests; the average total alkalinity among tanks varied by 5 μM (2239–2244 μM) and temperature by 0.4°C (11.2–11.6°C; S1 Fig, S1 Table). To examine which tanks differed from one another, *post hoc* Dunn’s pairwise joint ranking tests were performed for temperature and alkalinity. Among all tanks, temperature was distinct only between tanks in the low [O_2_] treatment; alkalinity was not distinct between tanks (S1 Fig, S1 Table). Abiotic differences between tanks in Experiment 2 did not measurably impact any of the biological response variables and as such were not considered biologically significant (see Results; S2 Fig).

**Fig 1:**
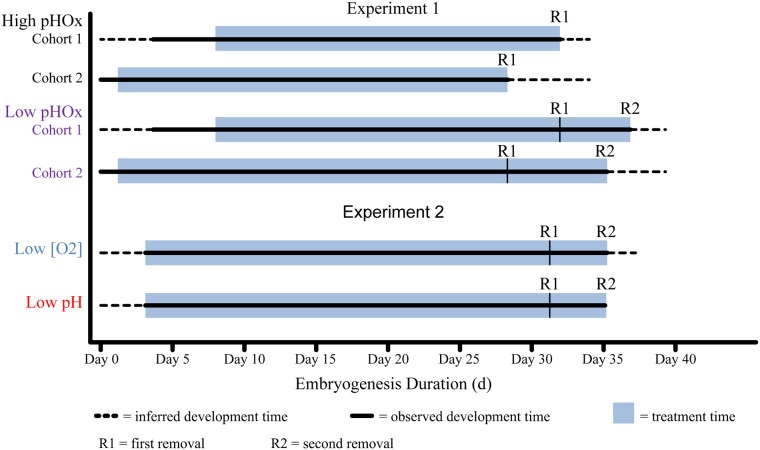
Exposure period for each treatment. *D*. *opalescens* capsule exposure duration (blue box) for each treatment. R indicates when embryo capsules were removed. R1 = first removal. R2 = second removal. Note: embryos from the High pHOx treatment were all collected at R1. Solid line = observed development time. Dotted line = inferred development time.

All capsules were exposed to their respective treatment for 24 days (d) or longer (Fig 1). Treatment impacts on development duration affected our experimental design: embryos from the low pHOx treatment took longer to develop than those from the high pHOx and were left in the treatment for an extended period (5–7 d). Therefore, half of the low pHOx treatment capsules were collected at the same time as all of the high pHOx treatment capsules with the other half of the low pHOx treatment collected later near hatching. Embryos from the Experiment 2 treatments (low pH only and low [O_2_] only) developed to the same stages over a similar duration of time as Experiment 1. Upon removal, embryo capsules were immediately photographed, and the total number of embryos per capsules was quantified. Then embryos were either preserved for morphological analyses or frozen for statolith analyses. A single capsule contains hundreds of embryo-filled chorions (usually one embryo per chorion), with each embryo having two statocysts and statoliths. Per capsule, approximately 50 embryos and their chorions, from the middle position of the capsule [34], were preserved for morphological measurement using formalin (5% formaldehyde in filtered seawater, 50 μm). Samples were taken from the middle positions to reduce developmental variation associated with a position within the capsule [34]. Near-hatch stages of embryos were frozen at -80°C and statoliths were later extracted and photographed for morphometric analyses.

**Fig 2:**
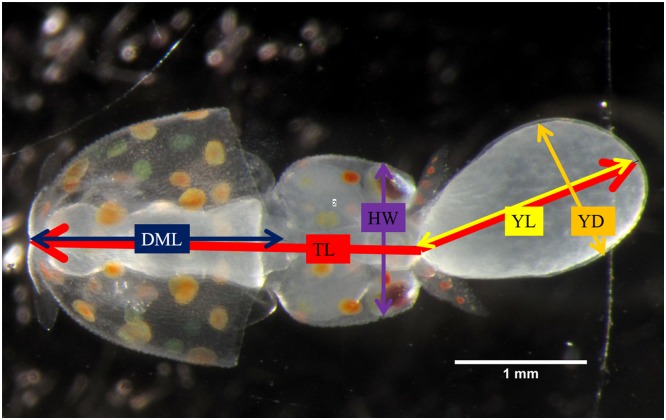
Embryo morphology. DML = Dorsal mantle length, TL = Total length, HW = Head width, YL = External yolk length, YD = External yolk diameter.

Embryo data were collected using photo microscopy (Canon PC1305, 4416 x 3312 pixels). Embryos were extracted from the chorion and photographed while flat with the dorsal side up. Embryo stages were determined using diagnostic, developmental features described for *D*. *pealii* by Arnold [30]. The dorsal side was identified based on the position of fins and, if the embryo was mature enough, chromatophore pattern. In addition to the embryo, the outer yolk was measured (referred to as “yolk”). Parameters measured included (1) total length of the embryo and yolk (TL), (2) head width (HW), (3) dorsal mantle length (DML), (4) yolk length (YL) and (5) yolk diameter (YD; Fig 2). Yolk length, height (h), diameter and radius (r) were used to estimate yolk volume (YV) as shown in the following equation:

$$YV=\left(\pi \;r^{2}\left(\frac{h}{3}\right)\right)+\;0.5\left(\left(\frac{4}{3}\right)\pi \;r^{3}\right)$$

where $r=\left(\frac{YD}{2}\right)$ and *h* = *YL* − *r*.

Embryo morphology metrics were measured using image processing and analysis through ImageJ 1.49v software.

Statoliths were dissected from the late developmental stage embryos (≥ stage 28) and removed from statocysts. Remaining tissue on statoliths was dissolved using a dilute bleach solution. Then, statoliths were rinsed three times with distilled water [27]. Statoliths were photographed via photo microscopy and measured by length, width, and area using ImageJ 1.47v software.

Treatment effects were first tested among embryos of the same age. Tanks were considered replicates for statistical testing of the hypotheses. Experiments 1 and 2 were tested separately. Embryo morphology data were Bonferroni corrected (ɑ = 0.0125) for a possible increase in type-1 errors from multiple testing. All data were first examined for variance homogeneity and tested for normality using residual analysis. To meet the normality assumption, YV, HW and TL data were log transformed in Experiment 2. All other morphometric data were normal without the need for transformation. Stage data was not normal, and these data were analyzed using Kruskal-Wallis tests and *post hoc* Dunn’s pairwise joint ranking tests.

For Experiment 1, since two cohorts were used, cohort effects were nested within treatment using a one-way hierarchal ANOVA [35]. Further, exposure effects were nested within each treatment and cohort tank effects within treatment, cohort and exposure group. Capsule effects were nested within each treatment, cohort, exposure group and tank. Capsule effects were considered “random” and treatment, cohort, tank, and exposure effects were considered “fixed.” In Experiment 2, a one-way hierarchal ANOVA was used to test for effects of treatment, exposure duration, tank, and capsule on embryonic structures. Exposure duration effects were nested within each treatment; tank effects were nested within each treatment and exposure group, and capsule effects were nested within each treatment, exposure group, and tank. Capsule effects were considered “random” and treatment, tank, and exposure effects were considered “fixed.”

A second analysis stratified treatment effects by embryo stage (rather than age). In addition to embryo data, statolith data were analyzed. All statolith data and embryo morphometric data from Experiment 1 were normal. However, embryo data from Experiment 2 were non normal. Experiments 1 and 2 were tested separately. For Experiment 1, embryos were tested for treatment effects by stage using a one-way hierarchal ANOVA. Cohort effects were nested within each treatment group, and capsule effects were nested within each treatment and cohort group. Stage, treatment and cohort effects were tested as “fixed” and capsule effect was tested as a “random” effect. Experiment 2 examined treatment effects by developmental stage using Wilcoxon tests. Effects of treatment on statolith structure were tested by one-way hierarchal ANOVA. Statoliths tested from Experiment 1 were all from cohort 1, embryo stage 28. Statoliths tested from Experiment 2 were from the 32-d exposure group and were all from embryo stage 29. The treatment effect was tested as “fixed” and capsule effect as “random.” Capsule effect was nested within the treatment effect. Statistical analyses were conducted using JMP software (Version 11 Pro).

## Results

**Fig 3:**
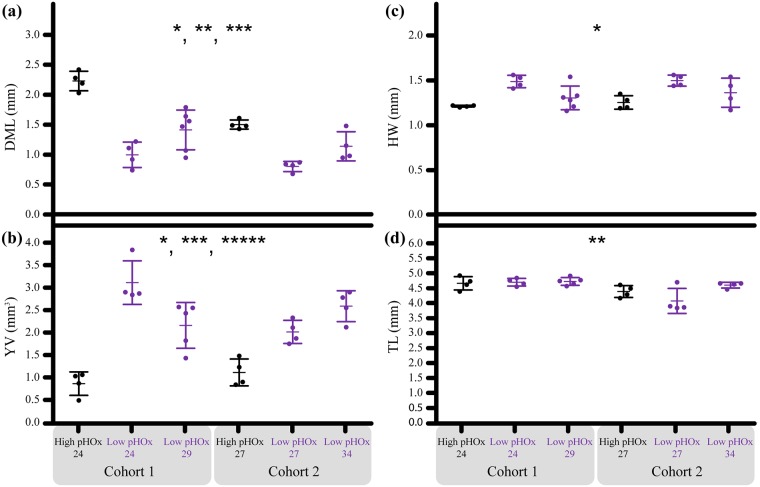
The embryonic response across treatments and exposure duration for Experiment 1. Each point represents the average embryo value per capsule (N = 10 embryos per capsule). X-axis = Numbers indicate the exposure duration in days. Points are grouped by treatment*exposure*cohort. (a) DML = Dorsal mantle length (mm). (b) YV = Yolk volume (mm^3^). (c) HW = Head width (mm). (d) TL = Total length (mm). * = treatment effect (***p < 0*.*001***), ** = cohort effect (***p < 0*.*0001***), *** = exposure duration effect (***p < 0*.*0125***), ***** = capsule effect (***p < 0*.*01***). There were no tank effects. Bars = ± 1 standard error.

Chronic exposure (≥ 24 d) to varied levels of pH and [O_2_] (pHOx) affect *D*. *opalescens* embryo development stage, yolk and statolith size. Embryos exposed to low pHOx had 149.9% larger YV, were 39.6% shorter in DML and had 13.0% wider HWs than those, of the same age exposed to high pHOx (Fig 3a and 3b). Cohort 1 was older and more developed than cohort 2 at removal (Fig 2). This stage advancement enhanced the low pHOx effect and Cohort 1 embryos exposed to low pHOx had 200% larger YVs, 44.1% shorter DMLs and 13.6% wider HWs compared to embryos of the same age exposed to high pHOx (Fig 3). The advanced development of cohort 1 relative to cohort 2 may indicate that embryos from cohort 1 were older when they were introduced into the experiment compared to those from cohort 2 (Fig 1).

In Experiment 1, there were no tank effects, but there were several nested effects (Fig 3; S2 Table). The two cohorts had distinct DMLs and TLs; cohort 1 embryos had 32.4% longer DMLs (*F*_2,25_ = 19.59, ***p < 0*.*0001*)** and 7.9% greater TLs (*F*_2,24_ = 8.96, ***p = 0*.*0043*)**. YV and HW were similar between cohorts. Embryos that were allowed more exposure time to the low pHOx treatment had smaller YVs compared to those with less exposure to low pHOx (*F*_4,24_ = 9.24, ***p = 0*.*0015***). Capsule effects also only significantly impacted embryo YV (*F*_16,24_ = 2.3142, ***p = 0*.*0086*)**.

**Fig 4:**
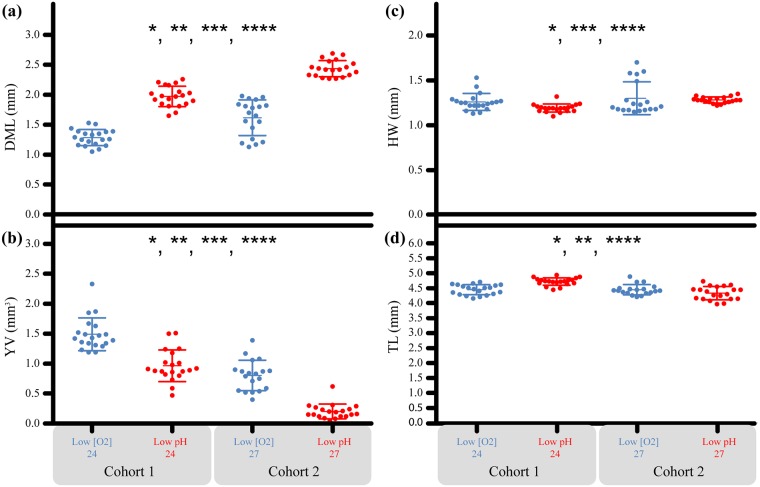
The embryonic response across treatments and exposure duration for Experiment 2. Each point represents the average embryo value per capsule (N = 10 embryos per capsule). X-axis: Numbers indicate exposure duration (days) and treatment is indicated as either low pH or low [O_2_]. (a) Y-axis = Dorsal mantle length (mm). (b) Y-Axis = Yolk volume (mm^3^). (c) Y-axis = Head width (mm). (d) Y-axis = Total length (mm). * = treatment effect (***p < 0*.*005***). ** = exposure duration effect (***p < 0*.*0001***), *** = tank effect (***p < 0*.*005***), **** = capsule effect (***p < 0*.*0001)***. Bars = ± 1 standard error.

**Fig 5:**
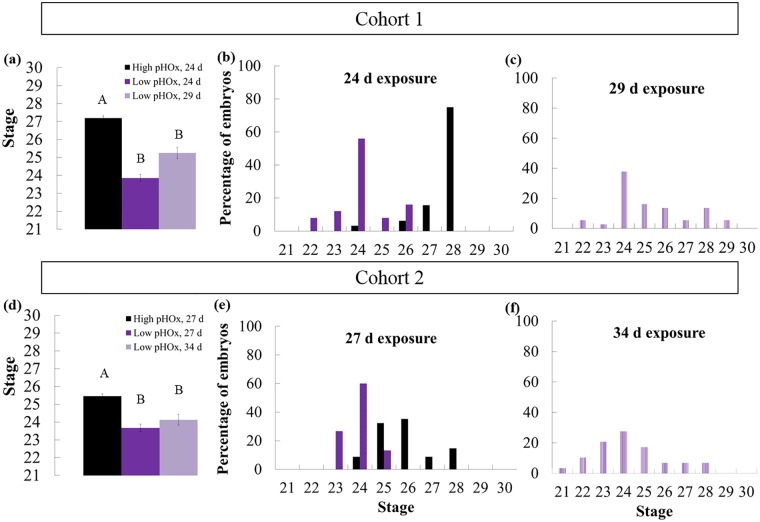
Experiment 1: Developmental categories of embryos across treatments and exposure duration. Black = Embryos from the High pHOx treatment, “standard” exposure duration, Dark Gray/Purple = Embryos from the Low pHOx treatment, “standard” exposure duration, Light Gray/Purple = Embryos from the Low pHOx treatment, “long” exposure duration. (a) Developmental categories of embryos from Cohort 1. Embryos were removed after 24 d (both treatments) and 29 d of exposure (low pHOx only). Treatments were significantly different (Kruskal-Wallis χ^2^ = 36.6724, DF = 2, ***p < 0*.*0001***) and letters indicate significant difference among groups (Dunn pair-wise joint ranking test, ***p < 0*.*0001***). Bar = ± 1 standard error. (b) Histogram of embryos exposed for 24 d to either high pHOx or low pHOx. (c) Histogram of embryos exposed for 29 d to low pHOx. (d) Developmental categories of embryos from Cohort 2. Embryos were removed at 27 d (both treatments) and 34 d (low pHOx only). Treatments were significantly different (Kruskal-Wallis χ^2^ = 28.0619, DF = 2, ***p < 0*.*0001***) and letters indicate significant difference among groups (Dunn pair-wise joint ranking test, ***p < 0*.*0001***). Bar = ± 1 standard error. (e) Histogram of embryos exposed for 27 d to either high pHOx or low pHOx. (f) Histogram of embryos exposed for 34 d to low pHOx.

**Fig 6:**
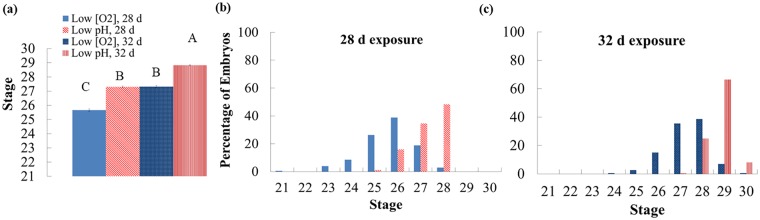
Experiment 2: Developmental categories of embryos across treatments and exposure duration. (a) Developmental categories of embryos removed from the experiment after 28 d of exposure to low [O_2_] (light solid) or low pH (diagonal stripes) and after 32 d of exposure to low [O_2_] (dark solid) or low pH (vertical stripes). Treatments were significantly different (Kruskal-Wallis χ^2^ = 472.8183, DF = 3, ***p < 0*.*0001***) and letters indicate significant difference among groups (Dunn pair-wise joint ranking test, ***p < 0*.*0001***). Bar = ± 1 standard error. (b) Histogram of embryos removed after 28 d of exposure to low [O_2_] (light solid) or low pH (diagonal stripes). (c) Histogram of embryos removed after 32 d of exposure to low [O_2_] (dark solid) or low pH (vertical stripes).

In Experiment 2, embryos exposed to low [O_2_] for 32 d were 19.1% shorter in DML and had 379.2% bigger yolks by volume compared to those, of the same age, from the low pH, 32-d treatment. Nearly all the outer yolk was absorbed by embryos from the low pH treatment. The embryos removed after 28 d of exposure from the low [O_2_] treatment had a 20.4% shorter dorsal mantle length and 85.9% larger yolk volume than those exposed to low pH for the same time duration (Fig 4a and 4b). These differences were significant (S2 Table) and correlated with developmental duration (Figs 5a–5f and 6a–6c).

Older embryos (32-d exposure) had longer DML (*F*_2,78_ = 253.79, ***p < 0*.*0001*)**, reduced YV (*F*_2,78_ = 143.09, ***p < 0*.*0001*)** and longer TL **(***F*_2,78_ = 14.79, ***p < 0*.*0001***). Exposure duration did not affect the HW between groups. For low pH treatment, the YV of the 32-d exposure group (0.201 mm^3^) approached zero and was reduced by 74.9% compared to the 28-d exposure group (0.801 mm^3^). For the low [O_2_] treatment 32-d exposure group, there was a 35.2% reduction of the YV (0.965 mm^3^) compared to embryos in the 28-d exposure group (1.490 mm^3^).

There was a tank effect in Experiment 2 for DML (*F*_4,78_ = 14.89, ***p < 0*.*0001*)**, YV (*F*_4,78_ = 13.39, ***p < 0*.*0001***) and HW (*F*_4,78_ = 4.11, ***p = 0*.*0047***), largely driven by differences between tanks in the pH treatment at 28 days of exposure. At 32 days, DML and HW were similar, and only YV was statistically distinct. Further, these biological tank effects did not mirror abiotic tank effects (S1 and S2 Figs) and therefore the abiotic differences are not driving them.

Capsules effects were observed for DML (*F*_71,78_ = 2.84, ***p < 0*.*0001*)**, HW (*F*_71,78_ = 5.69, ***p < 0*.*0001*)**, YV(*F*_71,78_ = 3.99, ***p < 0*.*0001*)**, and TL (*F*_71,78_ = 6.55, ***p < 0*.*0001*)**. These differences are biologically significant and are likely caused by the capsular and chorion membranes and maternal effects [25].

### Effects on Development

To quantify varied developmental duration and to identify age-independent treatment effects, embryos were compared by developmental stage [36]. Synchronous exposure to 7.55 pH (*p*CO_2_ = 1440 μatm) and 90 μM O_2_ affected embryos by slowing their development by on average 2.8 stages compared to all those exposed to the high pHOx treatment (Fig 5a–5c). For cohort 1, the low pHOx embryos were 3.5 stages behind those from the high pHOx treatment. With five to seven additional days of low pHOx exposure, some of these embryos reached as advanced stages as embryos from the high-pHOx treatment (Fig 5a, 5c, 5d and 5f). In contrast, cohort 2 low pHOx embryos were only 2.0 stages behind the high pHOx embryos and developed to similar advanced stages after seven additional days (Fig 5d–5f). In Experiment 2, embryos exposed to 84.7 μM O_2_ pooled from both time durations were on average 1.6 stages behind those exposed to low pH (Fig 6a–6c). Embryos in the low [O_2_] treatment were 1.7 and 1.5 stages behind those from the low-pH treatment for the 28-d and 32-d exposure groups, respectively.

**Fig 7:**
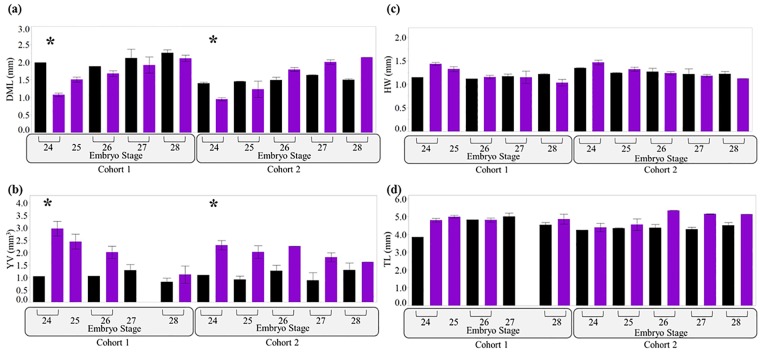
Experiment 1: Embryonic response to treatment when standardized by developmental stage. (a) DML = Dorsal mantle length (mm). (b) YV = Yolk volume (mm^3^). (c) HW = Head width (mm). (d) TL = Total length (mm). X-axis: Embryos are grouped by cohort, embryo stage and treatment. Numbers 24–28 = Embryo stage. Treatment = low pHOx (purple/gray) or high pHOx (black). * = treatment effect (***p < 0*.*001***). Bars = ± 1 standard error.

When comparing embryos from each treatment by embryo stage, all embryo size parameters (DML, YV, HW, and TL) at stages 25–28 were similar between low pHOx and high pHOx treatments in Experiment 1. Thus we infer that differences in size at age (Fig 3) are due to reduced growth rates (Fig 7). Interestingly, at stage 24, embryos from the low pHOx treatment were smaller (DML) and had larger yolks (Fig 7a and 7b). Overall, these data indicate that embryos that developed to stages > 25 in the low pHOx treatment were statistically similar in size compared to those from the high pHOx treatment (DML, YV, HW, TL; Fig 7; S3 Table). These embryos were morphologically resilient to chronic exposure to low environmental pHOx.

**Fig 8:**
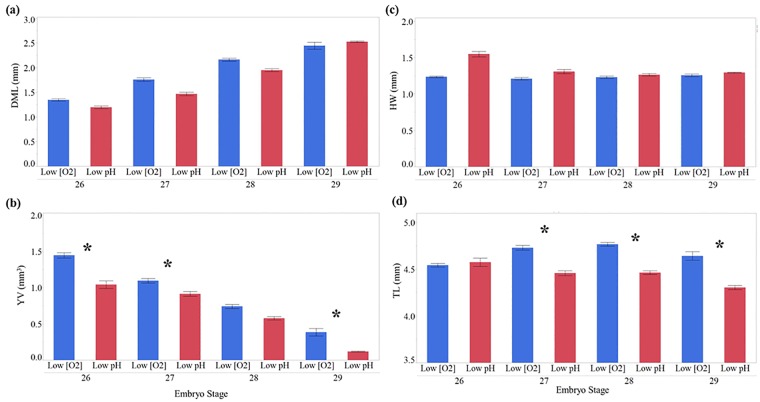
Experiment 2: Embryonic response to treatment when standardized by developmental category. (a) Y-axis = Dorsal mantle length (mm). (b) Y-Axis = Yolk volume (mm^3^). (c) HW = Head width (mm). (d) TL = Total length (mm). X-axis: Numbers 26–29 = Embryo stage. Treatment is indicated either low [O_2_] (blue/light gray) or low pH (red/dark gray). * = treatment effect (***p < 0*.*0125***). Bars = ± 1 standard error.

In Experiment 2, for most stages (26, 27 and 29) the embryos from the low pH treatment had a smaller yolk volume (Fig 8; S3 Table). Also, smaller yolks likely drive the pattern of these embryos having shorter TLs compared to those from the low [O_2_] group. DML and HW were similar between embryos from the low pH and low [O_2_] treatments.

**Fig 9:**
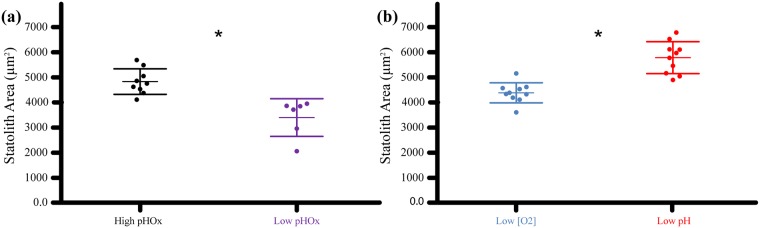
Embryonic statolith area (μm^2^) by treatment. Y-axis = average statolith area per capsule. Each capsule = Average of five statoliths. (a) Experiment 1. X-axis = treatment. All statoliths are from embryos at stage 28 in Cohort 1. * = treatment effect (***p = 0*.*0014***). (b) Experiment 2. X-axis = treatment. All statoliths are from embryos at stage 29. ** = treatment effect (***p < 0*.*0001***). Bars = ± 1 standard error.

We found statolith growth differences between treatments for both experiments. In Experiment 1, embryos at stage 28 in the low pHOx treatment had distinctly smaller statoliths, with 54.7% less area, 40.3% shorter length, and 7.3% smaller width than in the high pHOx treatment (Fig 9a). In Experiment 2, embryos had 31.9% smaller, 17.6% shorter, and 12.7% narrower statoliths in the low [O_2_] treatment compared to those from the same stage (29) in the low pH treatment (Fig 9b). Also, statoliths from the low pH/high *p*CO_2_ treatment did not have observable malformations and appeared normal.

## Discussion

This work reports the combined effects of environmental O_2_ and pH on embryonic squid development, duration, and growth. In some nearshore systems, including the California Current System, [O_2_] and pH naturally co-vary [37–38] over squid embryo habitat. The magnitude of biological response to the combined effects of these environmental factors warrants their inclusion into future laboratory study of squid development as well as for modeling environmental effects on squid embryos over their range.

### Embryo Development

We observed clear effects of exposure to chronic low pHOx on *D*. *opalescens* development, including increased developmental duration and decreased statolith size. A subset of these embryos exhibited malformations such as eye dimorphism and deformities in the mantle and body, similar to malformations observed in a congener as an embryonic response to elevated temperature [39]. Small embryonic size is a common response of marine invertebrates to low-environmental [O_2_] and is indicative of reduced growth rates [40–42]. In our study, neither low [O_2_] alone nor low pHOx resulted in decreased *D*. *opalescens* embryo size. Embryos from each capsule exposed to low pHOx treatment grew to similar sizes as embryos from the high pHOx treatment (except decreased statolith size). These embryos did not significantly differ from control embryos in size or yolk volume, suggesting that some squid embryos may be resilient to low pHOx conditions.

The coupling of pH and [O_2_] is important for embryo development. Embryos exposed to low pH had smaller external yolk sacs at each stage (26, 27, 29) compared to those from the low [O_2_] treatment, possibly indicating that these embryos were absorbing more yolk. This difference in YV suggests these embryos may be allocating more energy towards maintaining their internal pH balance relative to embryos exposed to low [O_2_]. Loliginid embryos exposed to acidified conditions have been shown to upregulate ATP-dependent ion pumps on the yolk epithelium to mediate extracellular pH regulation, suggesting a potential mechanism for elevated energy utilization in response to low pH [18, 43]. YV differences were not observed in Experiment 1. The YV morphology distinctions and the developmental duration differences of Experiment 2 demonstrate that coupling matters. Decoupling of the relationship between pH and [O_2_] could represent an extreme environmental change for the *D*. *opalescens* at the embryo life stage and pose serious challenge to their physiological limits. As such, future investigations not only should assess the magnitude for change of pH and [O_2_] under future climate change scenarios but should also assess whether or not the relationship between pH and [O_2_] will change from historic values.

Our results show that experimental exposure to low pHOx resulted in negative effects on statolith size and longer development durations for embryos at the same stage. The low pHOx effect could be additive resulting from the combining environmental stressors (pH, *p*CO_2_ and [O_2_]). During embryonic development, some cephalopods experience increased metabolic costs caused by additive effects of combined pH and *p*CO_2_ with another environmental stressor [28, 44–46]. However, in our experiments, no effects on embryo size were observed. The egg capsule surrounding cephalopod embryos acts as a diffusion barrier to gas exchange, resulting in naturally decreasing pH and [O_2_] over the course of development. As a result, cephalopods such as cuttlefish [46] and squid [28, 39] are tolerant to lower pH levels at temperatures near those in our study, and may be pre-adapted to coping with these conditions [47]. External seawater pH results in small relative pH change within the perivitelline fluid (PVF) of developing cephalopods [47], such as *D*. *opalescens* embryos. Although small, these additive changes represent a significant developmental challenge for embryos [14, 17, 44–45].

### Potential for Ecological Carry-Over Effects

Ecological carry-over effects occur when an individual’s previous history and experience explains their current performance in a given situation [48]. Potentially, an embryo’s developmental characteristics can affect its performance at later life stages. In this study, we found *D*. *opalescens* embryo size was stable as has been found with many embryos in response to environmental change [1]. Embryos must be large to optimize their hunting ability [49] and retain needed caloric reserves (yolk) to “buy” the time necessary to learn to hunt as paralarvae [50]. Embryo fitness at hatch and food availability determines which paralarvae survive the critical period to first feeding [50]. DML and YV size was independent of pHOx treatments in Experiment 1 suggesting that size is conserved. While embryo size was conserved, developmental duration and statolith size were impacted by [O_2_] and pH levels. As [O_2_] and pH naturally co-vary over squid egg beds [9], squid may experience altered development duration in response to changes in pHOx conditions. While this plasticity in development duration may allow squid to persist in a dynamic system, it is unknown whether corresponding hatching delays can cause a disruption in phenology due to the mismatch in timing of hatching and food availability. Downstream implications of our findings support the hypothesis that subsequent life stages of *D*. *opalescens* (e.g. paralarvae) exposed to low pHOx will be smaller than those, at the same age, exposed to high pHOx. Recently, *D*. *opalescens* paralarvae collected off of California were found to be smaller during La Niña years compared to paralarvae at the same age during El Niño years [51–52]. Squid exposed to low pHOx in nature may be impacted similarly as they are in the laboratory [51].

Impacts to sensory organs can greatly alter the survival rate of paralarvae [53]. Statocysts function as the gravity-sensing organ of the squid to detect acceleration in the x, y, and z-axes [23]. In our study, we found that statolith size was reduced for embryos from the low pHOx treatment relative to those from the high pHOx treatment. Another study, on *Doryteuthis pealeii*, found statoliths of embryos exposed to low environmental pH/high *p*CO_2_ (2200 μatm) treatments were significantly smaller than the control [27]. Statolith size changes, both positive and negative, can have dramatic effects on the fitness of the squid through changes in paralarvae behavior. Future research should measure statolith volume in addition to the morphometrics in this study. Specifically, decreases in any combination of length, area, and volume can reduce the ability of squid to sense jet/sink movements [23]. Furthermore, squid with impaired hunting ability have higher mortality rates at the paralarvae stage, especially through the critical period [50]. Our findings support the need for future studies on low pHOx impacts on downstream locomotion behavior (e.g. paralarvae sink/jet movements).

### Implications of Embryonic Squid Response to Low pHOx in Southern California

Low pHOx impacts squid embryogenesis duration and statolith size. Persistent upwelling events uplift oxypleths along the shelf of the Southern California Bight and similar uplifting occurs during La Niña [31]. Our laboratory results suggest that the best embryo habitat regarding pH, *p*CO_2_ and [O_2_] may be within the upper-shelf waters < 40 m depth, with harsher habitat occurring deeper. At shallow depths, semi-diurnal tidal currents usually bathe embryos attached to the seafloor with high [O_2_] and high pH/ low*p*CO_2_ waters [8, 9] promoting critical gas exchange during development (ventilation). If [O_2_] and pH levels in water masses are cues driving spawning site selection, *D*. *opalescens* might attach embryo capsules at different depths on the shelf at different times throughout the year [9]. Mapping the most persistently utilized embryo beds should be prioritized. Protection for these areas is likely warranted as squid utilize habitats that are higher in [O_2_] and pH that are predicted to shrink due to human-induced sources of hypoxia and hypercapnia [8].

## Supporting Information

S1 Data All Experiment 1 and 2 morphological data. (XLSX)

S1 Fig Tank effects on seawater properties. Seawater properties per tank. Box plot distribution of daily averages and variability for total alkalinity, temperature, pH and dissolved oxygen. A Kruskal-Wallis revealed tanks effects were statistically significant for temperature and total alkalinity (TA) in Experiment 2. *Post hoc* Dunn pair-wise joint ranking tests for tanks within treatment found that temperature was significant only between tanks in the low [O_2_] treatment. * = tank effect (Dunn’s Test; ***p = 0*.*0493***). Bars = ± 1 standard error.
(TIF)

S2 Fig Embryo morphology between [O_2_] tanks with distinct seawater temperature (S1 Fig). X-axis = [O_2_] tank replicates 1 and 2. Y-axis = Biological response variable. (a) Dorsal mantle length (mm) = DML. (b) Yolk volume (mm^3^) = YV. (c) Head width (mm) = HW. (d) Total length (mm) = TL. Bars = ± 1 standard error.
(TIF)

S1 Table Tank effects. Kruskal-Wallis test for tank effects and *post hoc* Dunn pair-wise joint ranking tests between tanks within each treatment (S1 Fig). (a) Experiment 1. (b) Experiment 2.
(DOCX)

S2 Table Treatment and nested effects on squid embryos. (a) Experiment 1, treatments = low pHOx, high pHOx. (a1) ANOVA results. (a2) *post hoc* TUKEY results (b) Experiment 2, treatments = low pH, low [O_2_]. (b1) ANOVA results. (b2) *post hoc* TUKEY results. DML = dorsal mantle length, YV = external yolk sac volume, HW = head width, TL = total length of the embryo and external yolk sac. All results were Bonferroni corrected (ɑ = 0.125). Bold and italicized font = significant.
(DOCX)

S3 Table Treatment effects across embryo stage categories. (a1) Experiment 1: Results of the hierarchal ANOVA. (a2) Experiment 1: *post hoc* Tukey test results. (b) Experiment 2: Results of the Wilcoxon test. DML = dorsal mantle length, YV = external yolk sac volume, HW = head width, TL = total length of the embryo and external yolk sac. All results were Bonferroni corrected (ɑ = 0.125). Bold and italicized font = significant.
(DOCX)
