# Supplementary figures and images for: Development of Embryonic Market Squid, Doryteuthis opalescens, under Chronic Exposure to Low Environmental pH and [O2]
Source: PLoS One. 2016 Dec 9;11(12):e0167461. doi: 10.1371/journal.pone.0167461 (PMC5147904; doi:10.1371/journal.pone.0167461)

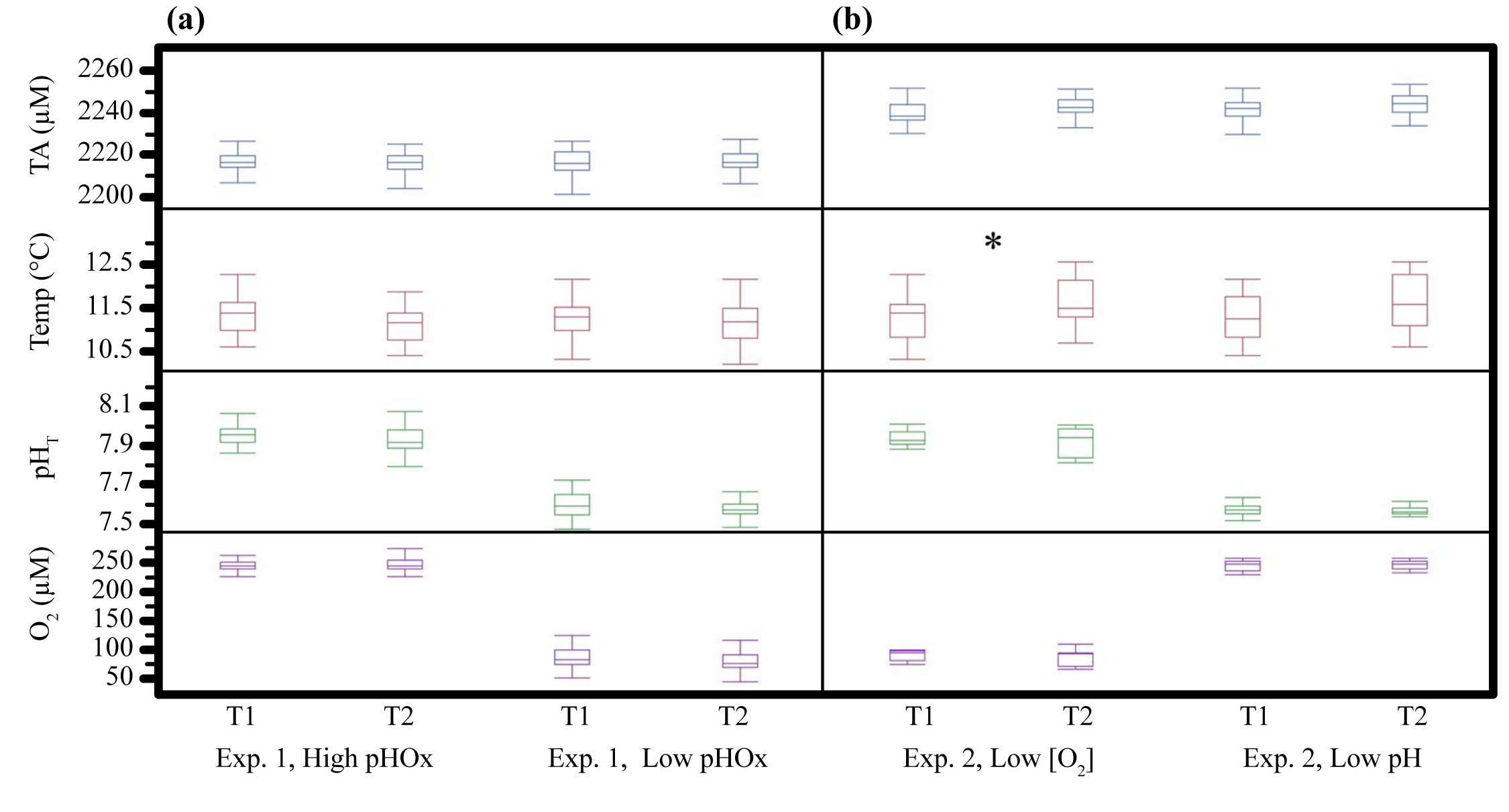

Supplement: S1 Fig — Seawater properties per tank. Box plot distribution of daily averages and variability for total alkalinity, temperature, pH and dissolved oxygen. A Kruskal-Wallis revealed tanks effects were statistically significant for temperature and total alkalinity (TA) in Experiment 2. Post hoc Dunn pair-wise joint ranking tests for tanks within treatment found that temperature was significant only between tanks in the low [O2] treatment. * = tank effect (Dunn’s Test; p = 0.0493). Bars = ± 1 standard error. (TIF) [file pone.0167461.s002.tif]

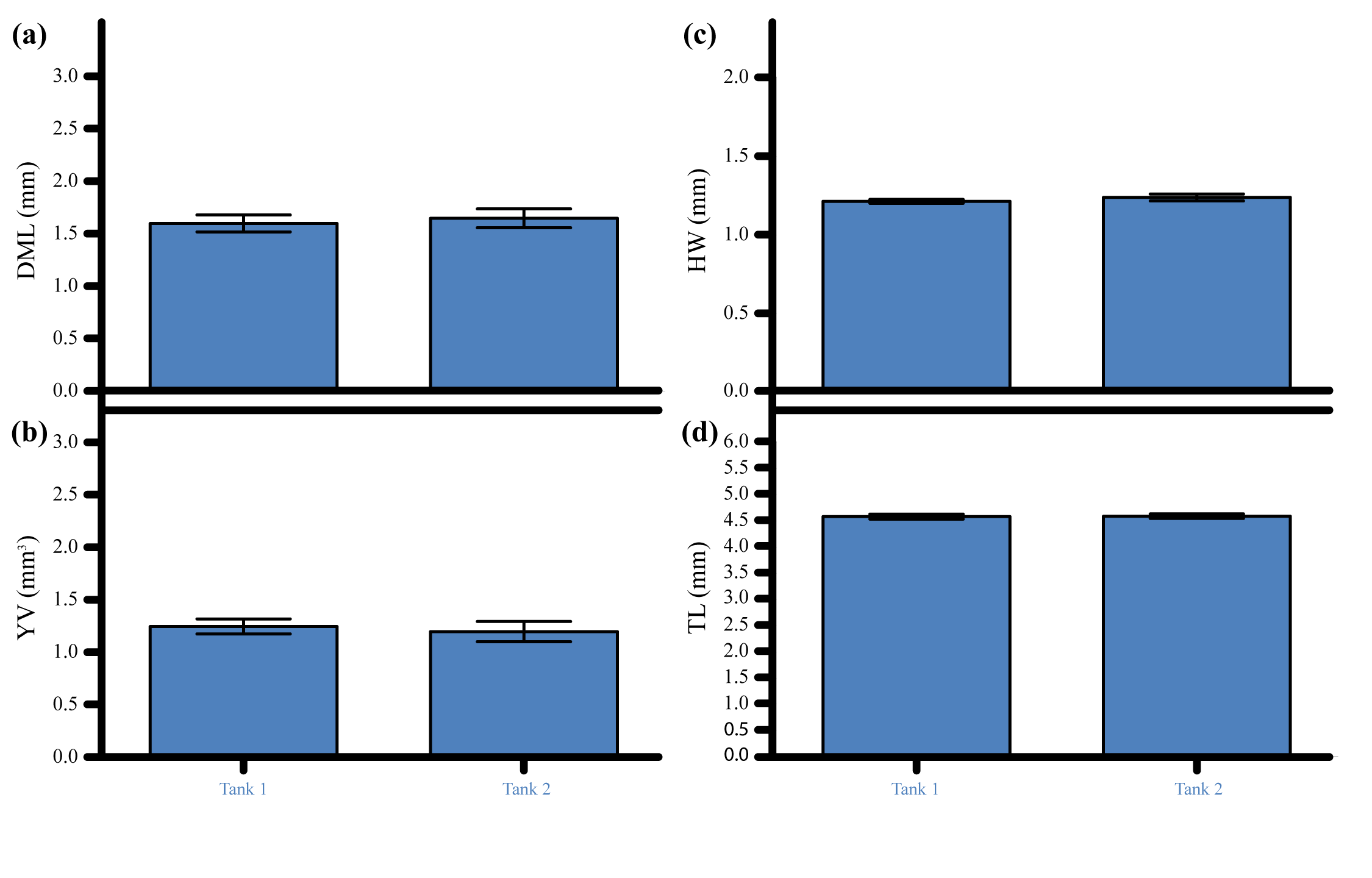

Supplement: S2 Fig — X-axis = [O2] tank replicates 1 and 2. Y-axis = Biological response variable. (a) Dorsal mantle length (mm) = DML. (b) Yolk volume (mm3) = YV. (c) Head width (mm) = HW. (d) Total length (mm) = TL. Bars = ± 1 standard error. (TIF) [file pone.0167461.s003.tif]
