# Supplementary material for: Development of Embryonic Market Squid, Doryteuthis opalescens, under Chronic Exposure to Low Environmental pH and [O2]
Source: PLoS One. 2016 Dec 9;11(12):e0167461. doi: 10.1371/journal.pone.0167461 (PMC5147904; doi:10.1371/journal.pone.0167461)
Supplement: S1 Table — Kruskal-Wallis test for tank effects and post hoc Dunn pair-wise joint ranking tests between tanks within each treatment (S1 Fig). (a) Experiment 1. (b) Experiment 2. (DOCX) [file pone.0167461.s004.docx]

**(a)**

**Experiment 1**

|  |  |  | **Low pHOx Dunn’s Test** | | **High pHOx Dunn’s Test** | |
| --- | --- | --- | --- | --- | --- | --- |
| **Variable χ2 (3, N = 37) p Z p Z p** | | | | | | |
| **Total Alkalinity (µM)** | 0.2964 | 0.9607 | 3.6952 | 1.00 | -3.703 | 1.00 |
| **Temperature (°C)** | 6.9250 | 0.0743 | -1.2970 | 1.00 | -2.2802 | 0.1356 |
| **pH** | 110.2690 | ***< 0.0001*** | -0.3243 | 1.00 | -0.9010 | 1.00 |
| **Oxygen (µM)** | 91.4550 | ***< 0.0001*** | -0.8003 | 1.00 | 0.2245 | 1.00 |

**(b)**

**Experiment 2**

|  |  |  | **Low [O_2_] Dunn’s Test** | | **Low pH Dunn’s Test** | |
| --- | --- | --- | --- | --- | --- | --- |
| **Variable χ2 (3, N = 33) p Z p Z p** | | | | | | |
| **Total Alkalinity (µM)** | 9.5939 | ***0.0224*** | 1.9860 | 0.2822 | 1.7164 | 0.5166 |
| **Temperature (°C)** | 12.5565 | ***0.0057*** | 2.6427 | ***0.0493*** | 2.3423 | 0.1150 |
| **pH** | 93.2027 | ***< 0.0001*** | 0.0138 | 1.00 | -0.4878 | 1.00 |
| **Oxygen (µM)** | 73.2092 | ***< 0.0001*** | -0.6808 | 1.00 | -0.0547 | 1.00 |
