# Supplementary material for: Development of Embryonic Market Squid, Doryteuthis opalescens, under Chronic Exposure to Low Environmental pH and [O2]
Source: PLoS One. 2016 Dec 9;11(12):e0167461. doi: 10.1371/journal.pone.0167461 (PMC5147904; doi:10.1371/journal.pone.0167461)
Supplement: S2 Table — (a) Experiment 1, treatments = low pHOx, high pHOx. (a1) ANOVA results. (a2) post hoc TUKEY results (b) Experiment 2, treatments = low pH, low [O2]. (b1) ANOVA results. (b2) post hoc TUKEY results. DML = dorsal mantle length, YV = external yolk sac volume, HW = head width, TL = total length of the embryo and external yolk sac. All results were Bonferroni corrected (ɑ = 0.125). Bold and italicized font = significant. (DOCX) [file pone.0167461.s005.docx]

**S2 Table. Treatment and nested effects on squid embryos.**

(a) Experiment 1, treatments = low pHOx, high pHOx. (a1) ANOVA results. (a2) *post hoc* TUKEY results (b) Experiment 2, treatments = low pH, low [O_2_]. (b1) ANOVA results. (b2) *post hoc* TUKEY results. DML = dorsal mantle length, YV = external yolk sac volume, HW = head width, TL = total length of the embryo and external yolk sac. All results were Bonferroni corrected (ɑ = 0.125). Bold and italicized font = significant.

(a1)

| **Variable Effect F (DF1, DF2) p** | | | |
| --- | --- | --- | --- |
| DML | Treatment | *F*_1,25_ = 80.48 | ***< 0.0001*** |
|  | Cohort | *F*_2,25_ = 19.60 | ***< 0.0001*** |
|  | Exposure Time | *F*_4,25_ = 5.29 | ***0.0118*** |
|  | Tank | *F*_8,25_ = 1.05 | 0.4354 |
|  | Capsule | *F*_16,25_ = 1.53 | 0.1062 |
| YV | Treatment | *F*_1,24_ = 92.38 | ***< 0.0001*** |
|  | Cohort | *F*_2,24_ = 1.51 | 0.2587 |
|  | Exposure Time | *F*_4,24_ = 9.24 | ***0.0015*** |
|  | Tank | *F*_8,24_ = 1.17 | 0.3769 |
|  | Capsule | *F*_16,24_ = 2.31 | ***0.0086*** |
| HW | Treatment | *F*_1,25_ = 18.98 | ***0.0006*** |
|  | Cohort | *F*_2,25_ = 0.35 | 0.7102 |
|  | Exposure Time | *F*_4,25_ = 4.43 | 0.0226 |
|  | Tank | *F*_8,25_ = 1.29 | 0.3214 |
|  | Capsule | *F*_16,25_ = 1.90 | 0.0312 |
| TL | Treatment | *F*_1,24_ = 0.002 | 0.9674 |
|  | Cohort | *F*_2,24_ = 8.96 | ***0.0043*** |
|  | Exposure Time | *F*_4,24_ = 4.44 | 0.0187 |
|  | Tank | *F*_8,24_ = 1.90 | 0.1520 |
|  | Capsule | *F*_16,24_ = 0.75 | 0.7076 |

(a2)

| **Effect** | **Level** | **DML** | **YV** | **HW** | **TL** |
| --- | --- | --- | --- | --- | --- |
| Cohort (C) | High pHOx C1 | ***A*** |  |  | AB |
|  | High pHOx C2 | ***B*** |  |  | A |
|  | Low pHOx C1 | CB |  |  | ***A*** |
|  | Low pHOx C2 | C |  |  | ***B*** |
| Exposure Duration (D) | Low pHOxC1 D24 | A | ***A*** |  |  |
|  | Low pHOxC1 D29 | AB | ***B*** |  |  |
|  | High pHOxC1 D24 | C | D |  |  |
|  | Low pHOxC2 D27 | A | BC |  |  |
|  | Low pHOxC2 D34 | A | BA |  |  |
|  | High pHOxC2 D27 | B | CD |  |  |
| Capsule | High pHOxC1D24T1 5 |  | I |  |  |
|  | High pHOxC1D24T1 7 |  | FGHI |  |  |
|  | High pHOxC1D24T2 2 |  | HI |  |  |
|  | High pHOxC1D24T2 3 |  | FGHI |  |  |
|  | Low pHOxC1D24T1 5 |  | AB |  |  |
|  | Low pHOxC1D24T1 7 |  | A |  |  |
|  | Low pHOxC1D24T2 5 |  | AB |  |  |
|  | Low pHOxC1D24T2 9 |  | ABCD |  |  |
|  | Low pHOxC1D29T1 2 |  | ***AB*** |  |  |
|  | Low pHOxC1D29T1 3 |  |  |  |  |
|  | Low pHOxC1D29T1 8 |  | ***CDEFGHI*** |  |  |
|  | Low pHOxC1D29T2 1 |  | ABCD |  |  |
|  | Low pHOxC1D29T2 3 |  | ABCDEFGH |  |  |
|  | Low pHOxC1D29T2 7 |  | ABCDEFGHI |  |  |
|  | High pHOxC2D27T1 4 |  | GHI |  |  |
|  | High pHOxC2D27T1 6 |  | EFGHI |  |  |
|  | High pHOxC2D27T2 1 |  | CEFGHI |  |  |
|  | High pHOxC2D27T2 4 |  | ABCDEFGHI |  |  |
|  | Low pHOxC2D27T1 1 |  | BCDEFGHI |  |  |
|  | Low pHOxC2D27T1 10 |  | HI |  |  |
|  | Low pHOxC2D27T2 6 |  | BCDEF |  |  |
|  | Low pHOxC2D27T2 8 |  | ABCDEFG |  |  |
|  | Low pHOxC2D34T1 4 |  | AB |  |  |
|  | Low pHOxC2D34T1 9 |  | AB |  |  |
|  | Low pHOxC2D34T2 2 |  | ABD |  |  |
|  | Low pHOxC2D34T2 4 |  | BCDE |  |  |

(b1)

| **Variable Effect F (DF1, DF2) p** | | | |
| --- | --- | --- | --- |
| DML | Treatment | F_1,78_ = 146.89 | ***< 0.0001*** |
|  | Exposure Time | F_2,78_ = 253.79 | ***< 0.0001*** |
|  | Tank | F_4,78_ = 14.89 | ***< 0.0001*** |
|  | Capsule | F_71,78_ = 2.84 | ***< 0.0001*** |
| YV | Treatment | F_1,78_ = 324.64 | ***< 0.0001*** |
|  | Exposure Time | F_2,78_ = 143.09 | ***< 0.0001*** |
|  | Tank | F_4,78_ = 13.39 | ***< 0.0001*** |
|  | Capsule | F_71,78_ = 3.99 | ***< 0.0001*** |
| HW | Treatment | F_1,78_ = 10.59 | ***0.0017*** |
|  | Exposure Time | F_2,78_ = 1.88 | 0.1595 |
|  | Tank | F_4,78_ = 4.11 | ***0.0047*** |
|  | Capsule | F_71,78_ = 5.69 | ***< 0.0001*** |
| TL | Treatment | F_1,78_ = 25.95 | ***< 0.0001*** |
|  | Exposure Time | F_2,78_ = 14.79 | ***< 0.0001*** |
|  | Tank | F_4,78_ = 2.33 | 0.0644 |
|  | Capsule | F_71,78_ = 6.55 | ***< 0.0001*** |

(b2)

| **Effect** | **Level** | **DML** | **YV** | **HW** | **TL** |
| --- | --- | --- | --- | --- | --- |
| Exposure Duration (D) | Low [O_2_] D28 | ***A*** | ***A*** |  | ***A*** |
|  | Low [O_2_] D32 | ***C*** | ***B*** |  | ***B*** |
|  | Low pH D28 | ***B*** | ***B*** |  | A |
|  | Low pH D32 | ***D*** | ***C*** |  | A |
| Tank  (T) | Low[O_2_] D28 T1 | A | A | A |  |
|  | Low [O_2_] D28 T2 | A | A | AB |  |
|  | Low pH D28 T1 | ***A*** | ***B*** | ***A*** |  |
|  | Low pH D28 T2 | ***B*** | ***C*** | ***B*** |  |
|  | Low [O_2_] D32 T1 | B | BA | B |  |
|  | Low [O_2_] D32 T2 | B | BC | B |  |
|  | Low pH D32 T1 | C | ***D*** | AB |  |
|  | Low pH D32 T2 | C | ***E*** | AB |  |
| Capsule | Low [O_2_]D28T1 1 | AB | ABCDEFGHI | FGHIJ | NOPQRSTUV |
|  | Low [O_2_]D28T1 13 | ABCD | ABCDEFG | DEFGHIJ | BCDEFGHIJKLMNOPQR |
|  | Low [O_2_]D28T1 21 | ABCDEFGH | ABCDE | IJ | ABCDEFGHIJKLMNOP |
|  | Low [O_2_]D28T1 24 | ABCDEFG | ABCDEF | IJ | HIJKLMNOPQRSTU |
|  | Low [O_2_]D28T1 42 | ABCD | ABCDE | FGHIJ | FGHIJKLMNOPQRST |
|  | Low [O_2_]D28T1 44 | AB | ABCD | ***BCDEFG*** | MNOPQRSTUV |
|  | Low [O_2_]D28T1 60 | ABCDEFGHIJ | ABCD | FGHIJ | CDEFGHIJKLMNOPQRST |
|  | Low [O_2_]D28T1 68 | ABCDE | ABCDEFGHIJ | EFGHIJ | HIJKLMNOPQRSTU |
|  | Low [O_2_]D28T1 87 | AB | ABCDEFG | FGHIJ | ***OPQRSTUV*** |
|  | Low [O_2_]D28T1 88 | ABCDEF | AB | EFGHIJ | ***ABCDEFGHIJK*** |
|  | Low [O_2_]D28T2 34 | A | A | DEFGHI | ABCDEFGHIJKLMNOP |
|  | Low [O_2_]D28T2 35 | ABCDEFGHI | ABCDE | FGHIJ | ABCDEFGHIJKLMNOPQR |
|  | Low [O_2_]D28T2 38 | ABCD | ABCDEFGHI | ***ABCD*** | ABCDEFGHIJKLMNO |
|  | Low [O_2_]D28T2 39 | ABCDEFGHIJKLM | ABCDEFGHIJ | FGHIJ | ABCDEFGHIJKLM |
|  | Low [O_2_]D28T2 53 | AB | ABCDEFGH | FGHIJ | KLMNOPQRSTU |
|  | Low [O_2_]D28T2 54 | A | ABC | DEFGHIJ | ***RSTUV*** |
|  | Low [O_2_]D28T2 74 | ABCDEFGHIJKLM | ABCDE | FGHIJ | ABCDEFGHIJKLMNO |
|  | Low [O_2_]D28T2 79 | ABCDEFG | ABCDEFG | FGHIJ | BCDEFGHIJKLMNOPQR |
|  | Low [O_2_]D28T2 85 | ABCDEFGHIJKL | ABCDEFGHIJKL | ABCDEF | ABCDEFGHIJKLMNOP |
|  | Low pHD28T1 5 | ABCDEFGHIJKLMN | DEFGHIJKLMN | FGHIJ | ABCDE |
|  | Low pHD28T1 10 | ABCDEFGHIJKLMNO | ABCDEFGHIJ | FGHIJ | ABC |
|  | Low pHD28T1 25 | BCDEFGHIJKLMNO | BCDEFGHIJKL | FGHIJ | ABCDEFGHI |
|  | Low pHD28T1 29 | ABCDEFGHIJK | BCDEFGHIJKL | FGHIJ | ABCDEFGHIJKLMN |
|  | Low pHD28T1 46 | ABCD | BCDEFGHIJKL | FGHIJ | ABCDEF |
|  | Low pHD28T1 59 | ABC | BCDEFGHIJKL | FGHIJ | ABCDEFGHIJ |
|  | Low pHD28T1 63 | ***A*** | ABCDEFGHIJ | FGHIJ | ABCDEFGHIJKLMNOPQ |
|  | Low pHD28T1 66 | ***AB*** | BCDEFGHIJKL | FGHIJ | A |
|  | Low pHD28T1 67 | CDEFGHIJKLMNOP | ABCDE | FGHIJ | ABCDEFGHIJK |
|  | Low pHD28T1 8 | ABCD | ABCDEFGHIJ | FGHIJ | ABCDEFGH |
|  | Low pHD28T2 2 | FGHIJKLMNOPQRS | ABCDEFGHIJKL | FGHIJ | ABCDEFGH |
|  | Low pHD28T2 6 | LMNOPQRSTUVWX | ABCDEFGHIJK | GHIJ | ABCDEFGHIJ |
|  | Low pHD28T2 19 | EFGHIJKLMNOPQR | BCDEFGHIJKL | FGHIJ | ABCDEFGH |
|  | Low pHD28T2 32 | HIJKLMNOPQRSTUV | BCDEFGHIJKL | FGHIJ | ABCDEF |
|  | Low pHD28T2 37 | EFGHIJKLMNOPQ | ABCDEFGHIJKL | HIJ | ABCDEFGHIJKL |
|  | Low pHD28T2 50 | JKLMNOPQRSTUV | BCDEFGHIJKLM | J | EFGHIJKLMNOPQRST |
|  | Low pHD28T2 70 | KLMNOPQRSTUVW | BCDEFGHIJKL | FGHIJ | ABCDEFG |
|  | Low pHD28T2 80 | FGHIJKLMNOPQR | ABCDE | FGHIJ | ***ABCD*** |
|  | Low pHD28T2 83 | FGHIJKLMNOPQRST | ***IJKLMNO*** | FGHIJ | ABCDEFGHIJKL |
|  | Low [O_2_]D32T1 20 | GHIJKLMNOPQRST | BCDEFGHIJKL | FGHIJ | FGHIJKLMNOPQRST |
|  | Low [O_2_]D32T1 23 | LMNOPQRSTUVWX | BCDEFGHIJKL | FGHIJ | ABCDEFGHIJKLMNOP |
|  | Low [O_2_]D32T1 26 | KLMNOPQRSTUVWX | ABCDEFGHIJ | FGHIJ | FGHIJKLMNOPQRST |
|  | Low [O_2_]D32T1 27 | IJKLMNOPQRSTU | BCDEFGHIJKL | ABC | FGHIJKLMNOPQRST |
|  | Low [O_2_]D32T1 51 | QRSTUVWXYZ | ABCDEFGHIJ | FGHIJ | HIJKLMNOPQRST |
|  | Low [O_2_]D32T1 52 | NOPQRSTUVWXY | ABCDEFG | ***A*** | ***AB*** |
|  | Low [O_2_]D32T1 57 | ***BCDEFGHIJKLMNO*** | BCDEFGHIJKL | ***AB*** | HIJKLMNOPQRST |
|  | Low [O_2_]D32T1 61 | PQRSTUVWXYZ | BCDEFGHIJKL | ***AB*** | EFGHIJKLMNOPQRST |
|  | Low [O_2_]D32T1 76 | ***DEFGHIJKLMNOP*** | CDEFGHIJKLM | FGHIJ | CDEFGHIJKLMNOPQRST |
|  | Low [O_2_]D32T1 89 | FGHIJKLMNOPQR | BCDEFGHIJKL | ABCDE | BCDEFGHIJKLMNOPQRS |
|  | Low [O_2_]D32T2 30 | OPQRSTUVWXY | HIJKLMNO | FGHIJ | IJKLMNOPQRSTU |
|  | Low [O_2_]D32T2 33 | JKLMNOPQRSTUVWX | JKLMNO | FGHIJ | OPQRSTUV |
|  | Low [O_2_]D32T2 36 | IJKLMNOPQRSTUV | BCDEFGHIJKL | EFGHIJ | ***ABCDEFGHIJK*** |
|  | Low [O_2_]D32T2 40 | MNOPQRSTUVWXY | GHIJKLMNO | EFGHIJ | CDEFGHIJKLMNOPQRST |
|  | Low [O_2_]D32T2 41 | FGHIJKLMNOPQR | JKLMNO | FGHIJ | JKLMNOPQRSTU |
|  | Low [O_2_]D32T2 58 | JKLMNOPQRSTUV | BCDEFGHIJKL | FGHIJ | ***ABCDEFGHIJK*** |
|  | Low [O_2_]D32T2 64 | QRSTUVWXYZ | LMNOPQ | FGHIJ | MNOPQRSTUV |
|  | Low [O_2_]D32T2 78 | GHIJKLMNOPQRST | KLMNOP | FGHIJ | PQRSTUV |
|  | Low [O_2_]D32T2 82 | PQRSTUVWXYZ | EFGHIJKLMN | FGHIJ | FGHIJKLMNOPQRST |
|  | Low pHD32T1 3 | VWXYZ | RSTUVWX | FGHIJ | ***OPQRSTUV*** |
|  | Low pHD32T1 4 | VWXYZ | ***MNOPQR*** | EFGHIJ | ABCDEFGHIJKLMNOP |
|  | Low pHD32T1 7 | QRSTUVWXYZ | OPQRST | DEFGHIJ | ***MNOPQRSTUV*** |
|  | Low pHD32T1 14 | VWXYZ | QRSTUVWX | EFGHIJ | ABCDEFGHIJKLMNOP |
|  | Low pHD32T1 15 | TUVWXYZ | PQRSTU | FGHIJ | ***LMNOPQRSTUV*** |
|  | Low pHD32T1 22 | QRSTUVWXYZ | NOPQRS | FGHIJ | EFGHIJKLMNOPQRST |
|  | Low pHD32T1 55 | UVWXYZ | STUVWXY | EFGHIJ | ***RSTUV*** |
|  | Low pHD32T1 56 | QRSTUVWXYZ | ***FGHIJKLMN*** | DEFGH | ABCDEFGHI |
|  | Low pHD32T1 72 | VWXYZ | PQRSTUVW | EFGHIJ | ABCDEFGHIJKLMNOP |
|  | Low pHD32T1 77 | STUVWXYZ | NOPQRS | DEFGHIJ | FGHIJKLMNOPQRST |
|  | Low pHD32T2 9 | Z | ***Y*** | DEFGHIJ | UV |
|  | Low pHD32T2 16 | QRSTUVWXYZ | RSTUVWXY | DEFGHIJ | TUV |
|  | Low pHD32T2 28 | Z | VWXY | BCDEFGH | ***DEFGHIJKLMNOPQRST*** |
|  | Low pHD32T2 31 | YZ | ***PQRSTUV*** | CDEFGH | ***ABCDEFGHIJKLMNO*** |
|  | Low pHD32T2 48 | Z | QRSTUVWX | DEFGHI | ***EFGHIJKLMNOPQRST*** |
|  | Low pHD32T2 62 | Z | UVWXY | DEFGHI | ***GHIJKLMNOPQRST*** |
|  | Low pHD32T2 69 | Z | TUVWXY | DEFGHI | STUV |
|  | Low pHD32T2 75 | WXYZ | UVWXY | DEFGHIJ | RSTUV |
|  | Low pHD32T2 81 | XYZ | WXY | DEFGHI | QRSTUV |
|  | Low pHD32T2 90 | QRSTUVWXYZ | XY | FGHIJ | V |
