# Supplementary material for: Development of Embryonic Market Squid, Doryteuthis opalescens, under Chronic Exposure to Low Environmental pH and [O2]
Source: PLoS One. 2016 Dec 9;11(12):e0167461. doi: 10.1371/journal.pone.0167461 (PMC5147904; doi:10.1371/journal.pone.0167461)
Supplement: S3 Table — (a1) Experiment 1: Results of the hierarchal ANOVA. (a2) Experiment 1: post hoc Tukey test results. (b) Experiment 2: Results of the Wilcoxon test. DML = dorsal mantle length, YV = external yolk sac volume, HW = head width, TL = total length of the embryo and external yolk sac. All results were Bonferroni corrected (ɑ = 0.125). Bold and italicized font = significant. (DOCX) [file pone.0167461.s006.docx]

**S3 Table. Treatment effects across embryo stage categories.**

(a1) Experiment 1: Results of the hierarchal ANOVA. (a2) Experiment 1: *post hoc* Tukey test results. (b) Experiment 2: Results of the Wilcoxon test. DML = dorsal mantle length, YV = external yolk sac volume, HW = head width, TL = total length of the embryo and external yolk sac. All results were Bonferroni corrected (ɑ = 0.125). Bold and italicized font = significant.

(a1)

| **Variable Effect F (DF1, DF2) p** | | | |
| --- | --- | --- | --- |
| DML | Stage | *F*_4,53_ = 18.5046 | ***0.0001*** |
|  | Treatment | *F*_5,53_ = 5.4276 | ***0.0004*** |
|  | Cohort | *F*_9,53_ = 6.5676 | ***< 0.0001*** |
|  | Capsule | *F*_35,53_ = 2.9867 | ***< 0.0001*** |
| YV | Stage | *F*_4,47_ = 1.40005 | 0.2479 |
|  | Treatment | *F*_5,47_ = 6.2274 | ***0.0002*** |
|  | Cohort | *F*_8,47_ = 1.2667 | 0.2868 |
|  | Capsule | *F*_30,47_ = 2.5515 | ***0.0004*** |
| HW | Stage | *F*_4,53_ = 4.0442 | ***0.0054*** |
|  | Treatment | *F*_5,53_ = 2.0496 | 0.0833 |
|  | Cohort | *F*_9,53_ = 0.3508 | 0.7102 |
|  | Capsule | *F*_35,53_ = 1.8661, | ***0.0095*** |
| TL | Stage | *F*_4,47_ = 1.8913 | 0.1234 |
|  | Treatment | *F*_5,47_ = 2.1665 | 0.0696 |
|  | Cohort | *F*_8,47_ = 1.4550 | 0.1978 |
|  | Capsule | *F*_30,47_ = 1.5151 | 0.0720 |

(a2)

| **Effect** | **Level** | **DML** | **YV** | **HW** |
| --- | --- | --- | --- | --- |
| Stage (S) | 24 | ***A*** |  | ***A*** |
|  | 25 | ***A*** |  | AB |
|  | 26 | ***B*** |  | AB |
|  | 27 | BC |  | AB |
|  | 28 | ***C*** |  | ***B*** |
| Treatment | S24LowpHOx | ***A*** | ***A*** |  |
|  | S24HighpHOx | ***BCD*** | ***BC*** |  |
|  | S25LowpHOx | B | AB |  |
|  | S25HighpHOx | BC | BC |  |
|  | S26LowpHOx | BCD | ABC |  |
|  | S26HighpHOx | BCD | BC |  |
|  | S27LowpHOx | CD | ABC |  |
|  | S27HighpHOx | CD | C |  |
|  | S28LowpHOx | D | BC |  |
|  | S28HighpHOx | CD | C |  |
| Cohort (C) | S24LowpHOx C1 | FG |  |  |
|  | S24LowpHOx C2 | G |  |  |
|  | S24HighpHOx C1 | ABCDEF |  |  |
|  | S24HighpHOx C2 | BCDEFG |  |  |
|  | S25LowpHOx C1 | CDE |  |  |
|  | S25LowpHOx C2 | EFG |  |  |
|  | S25HighpHOx C1 |  |  |  |
|  | S25HighpHOx C2 | BCDEFG |  |  |
|  | S26LowpHOx C1 | BCDE |  |  |
|  | S26LowpHOx C2 | ABCDE |  |  |
|  | S26HighpHOx C1 | ABCDE |  |  |
|  | S26HighpHOx C2 | DE |  |  |
|  | S27LowpHOx C1 | ABCDE |  |  |
|  | S27LowpHOx C2 | ABCD |  |  |
|  | S27HighpHOx C1 | ABC |  |  |
|  | S27HighpHOx C2 | BCDEF |  |  |
|  | S28LowpHOx C1 | AB |  |  |
|  | S28LowpHOx C2 | ABCD |  |  |
|  | S28HighpHOx C1 | ***A*** |  |  |
|  | S28HighpHOx C2 | ***BCDEF*** |  |  |
| Capsule | S24HighpHOxC1 5 | ABCDEFGHI | CDEFGHIJ | ABCDE |
|  | S24HighpHOxC2 4 | EFGHIJKLMNOPQR |  | ABCDE |
|  | S24HighpHOxC2 6 | GHIJKLMNOPQR | EFGHIJ | ABCDE |
|  | S24LowpHOxC1 1 | LMNOPQR | A | ABCDE |
|  | S24LowpHOxC1 2 | MOP | ABCDE | ABCDE |
|  | S24LowpHOxC1 3 | JKLMNOPQR | ABCDEFGHI | ABCDE |
| Capsule  (cont.) | **Level** | **DML** | **YV** | **HW** |
|  | S24LowpHOxC1 5 | P | ABD | AD |
|  | S24LowpHOxC1 7 | OP | AB | ABD |
|  | S24LowpHOxC1 8 | IJKLMNOPQR | ABCDEFGHIJ | ABCDE |
|  | S24LowpHOxC1 9 | IJKLMNOPQR | ABCDEF | ABCDE |
|  | S24LowpHOxC2 10 | P | BCDEFGHIJ | ABCDE |
|  | S24LowpHOxC2 4 | OP | ABCD | ABCDE |
|  | S24LowpHOxC2 6 | OP | ABCDEFGHI | A |
|  | S24LowpHOxC2 8 | KLMNOPQR | ABCDEFGI | ABCD |
|  | S24LowpHOxC2 9 | MOPR | ABCDEFGHI | ABCDE |
|  | S25HighpHOxC2 4 | HIJKLNQR | GHJ | BCE |
|  | S25HighpHOxC2 6 | EFGHIJKLMNOPQR | CEFGHIJ | ABCDE |
|  | S25LowpHOxC1 1 | DEFGHIJKL | ABCDEFGHI | ABCDE |
|  | S25LowpHOxC1 3 | EFGHIJKLMNOPQR | ABCDEFGHIJ | ABCDE |
|  | S25LowpHOxC1 5 | EFGHIJKLMNOPQR | ABCDEFGI | ABCDE |
|  | S25LowpHOxC1 8 | EFGHIJK | EFGHIJ | BCDE |
|  | S25LowpHOxC1 9 | FGHIJKLMNOPQR | ABCDEFGHI | ABCDE |
|  | S25LowpHOxC2 1 | MNOPQR | ABCDEFGHIJ | ABCDE |
|  | S25LowpHOxC2 4 | GHIJKLNQ | BCDEFGHIJ | ABCDE |
|  | S25LowpHOxC2 6 | MOPQR | ABCDEFGHIJ | ABCDE |
|  | S25LowpHOxC2 9 | ABCDEFGHIJKLMNQR | ABCDEFGHI | ABCDE |
|  | S26HighpHOxC1 7 | BCDEFGH | EFGHIJ | ABCDE |
|  | S26HighpHOxC2 1 | FGHIJ | FGHIJ | ABCDE |
|  | S26HighpHOxC2 4 | GHIJKLMNOPQR | BCDEFGHIJ | ABCDE |
|  | S26HighpHOxC2 6 | GHIJKLMNQR | GHJ | CE |
|  | S26LowpHOxC1 1 | BCDEFGHI | ABCDEFGHIJ | BCDE |
|  | S26LowpHOxC1 3 | DEFGHIJK |  | ABCDE |
|  | S26LowpHOxC1 5 | GHIJKLNQR | ABCDEFI | ABCDE |
|  | S26LowpHOxC1 8 | DEFGHIJKLMNOPQR | ABCDEFGHIJ | ABCDE |
|  | S26LowpHOxC2 2 | ABCDEFGHIJKL | ABCDEFGHIJ | ABCDE |
|  | S26LowpHOxC2 4 | ABCDEFGHIJKLMNQR |  | ABCDE |
|  | S27HighpHOxC1 2 | ABCDEFGHIJK | ABCDEFGHIJ | ABCDE |
|  | S27HighpHOxC1 3 | AB | GHIJ | BCDE |
|  | S27HighpHOxC2 4 | CDEFGHIJKLMNOQR | BCDEFGHIJ | ABCDE |
|  | S27HighpHOxC2 6 | EFGHIJKLN | HJ | CE |
|  | S27LowpHOxC1 1 | ABCDEFG |  | ABCDE |
|  | S27LowpHOxC1 2 | BCDEFGHIJKLMNQR |  | BCDE |
|  | S27LowpHOxC2 2 | ABCDEFGHIJ | ABCDEFGHIJ | ABCDE |
|  | S27LowpHOxC2 9 | ABCDEFGH | ABCDEFGHIJ | ABCDE |
|  | S28HighpHOxC1 2 | ABCD | HJ | BCE |
|  | S28HighpHOxC1 3 | A | GHIJ | CE |
|  | S28HighpHOxC1 5 | ABC | J | CE |
|  | S28HighpHOxC1 7 | ABCDE | GHJ | BCE |
|  | S28HighpHOxC2 1 | FGHIJKLMNOQR | EFGHIJ | ABCDE |
|  | S28HighpHOxC2 4 | GHIJKLMNQR | CEFGHIJ | ABCDE |
|  | **Level** | **DML** | **YV** | **HW** |
|  | S28LowpHOxC1 1 | ABCD | ABCDEFGHIJ | CE |
|  | S28LowpHOxC1 3 | ABCDEFGH |  | BCDE |
|  | S28LowpHOxC1 8 | ABCDEFGHI | CEFGHIJ | E |
|  | S28LowpHOxC2 2 | ABCDEF | BCDEFGHIJ | BCE |

(b)

| **Variable Effect Stage χ^2^ (DF, N) p** | | | | |
| --- | --- | --- | --- | --- |
| DML | Treatment | 26 | **χ^2^** _(1, N = 39)_ = 3.6222 | 0.0570 |
|  |  | 27 | **χ^2^** _(1, N = 45)_ = 2.4972 | 0.1140 |
|  |  | 28 | **χ^2^** _(1, N = 47)_ = 2.4557 | 0.1171 |
|  |  | 29 | **χ^2^** _(1, N = 22)_ = 0.1812 | 0.6704 |
| YV | Treatment | 26 | **χ^2^** _(1, N = 39)_ = 7.0403 | ***0.0080*** |
|  |  | 27 | **χ^2^** _(1, N = 45)_ = 8.9103 | ***0.0028*** |
|  |  | 28 | **χ^2^** _(1, N = 47)_ = 5.2047 | 0.0225 |
|  |  | 29 | **χ^2^** _(1, N = 22)_ = 3.02196 | ***0.0022*** |
| HW | Treatment | 26 | **χ^2^** _(1, N = 39)_ = 5.9269 | 0.0149 |
|  |  | 27 | **χ^2^** _(1, N = 45)_ = 1.5398 | 0.2147 |
|  |  | 28 | **χ^2^** _(1, N = 47)_ = 0.0551 | 0.8145 |
|  |  | 29 | **χ^2^** _(1, N = 22)_ = 0.2609 | 0.6095 |
| TL | Treatment | 26 | **χ^2^** _(1, N = 39)_ = 0.0335 | 0.8548 |
|  |  | 27 | **χ^2^** _(1, N = 45)_ = 8.2914 | ***0.0041*** |
|  |  | 28 | **χ^2^** _(1, N = 47)_ = 15.642 | ***< 0.0001*** |
|  |  | 29 | **χ^2^** _(1, N = 22)_ = 7.4203 | ***0.0064*** |
